# Supplementary material for: Molecular evolution of the mammalian kinetochore complex
Source: bioRxiv. 2024 Jun 27:2024.06.27.600994. Preprint. [Version 1] doi: 10.1101/2024.06.27.600994 (PMC11230421; doi:10.1101/2024.06.27.600994)
Supplement: Supplement 5 [file NIHPP2024.06.27.600994v1-supplement-5.pdf]

bioRxiv preprint doi: https://doi.org/10.1101/2024.06.27.600994; this version posted June 27, 2024. The copyright holder for this preprint (which was not certified by peer review) is the author/funder, who has granted bioRxiv a license to display the preprint in perpetuity. It is made available under aCC-BY 4.0 International license.

the models, and the vertical bars represent the number of proteins that are significant under the specified subset of models, as indicated by the dots below.

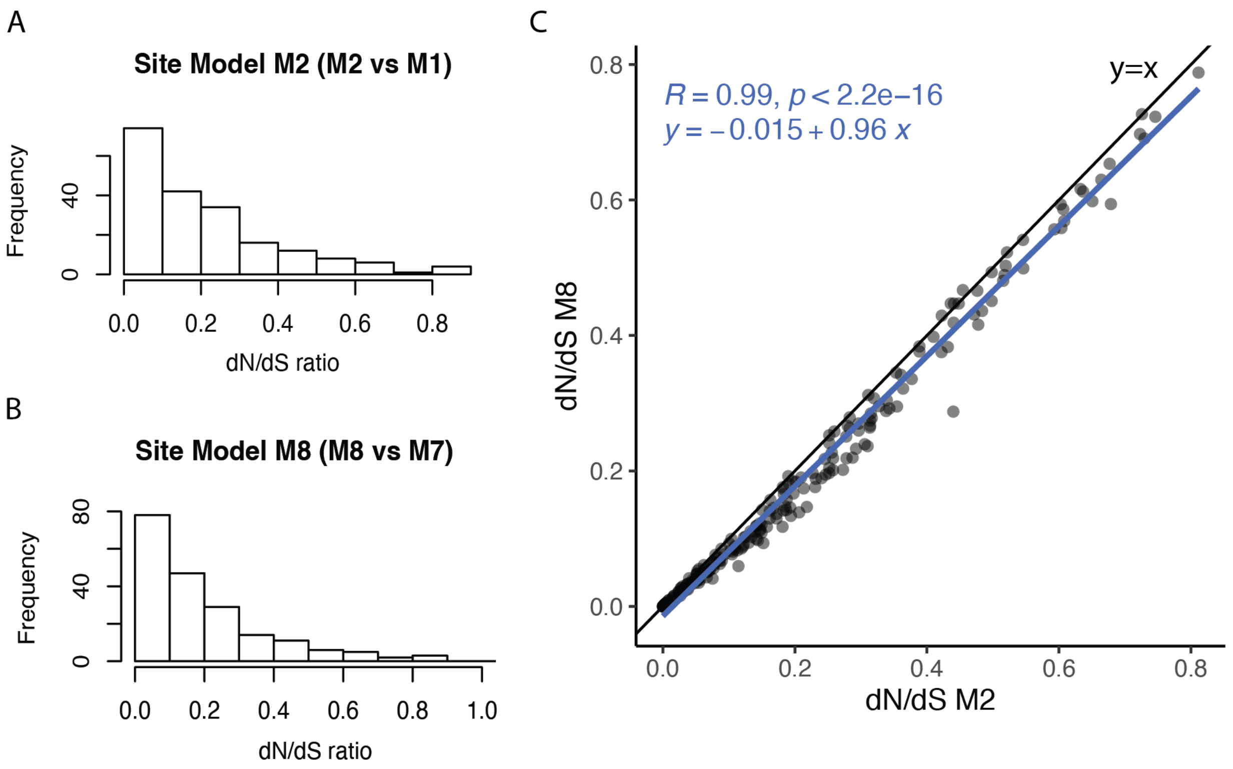

Supplementary Figure 1: **Majority of kinetochore proteins evolve under purifying selection** (A and B) Histograms representing dN/dS ratios (ω) of kinetochore proteins estimated under two implementations of the site model: (A) M2 and (B) M8. (C) dN/dS (ω) values under the M2 and M8 frameworks are strongly positively correlated.

bioRxiv preprint doi: https://doi.org/10.1101/2024.06.27.600994; this version posted June 27, 2024. The copyright holder for this preprint (which was not certified by peer review) is the author/funder, who has granted bioRxiv a license to display the preprint in perpetuity. It is made available under aCC-BY 4.0 International license.

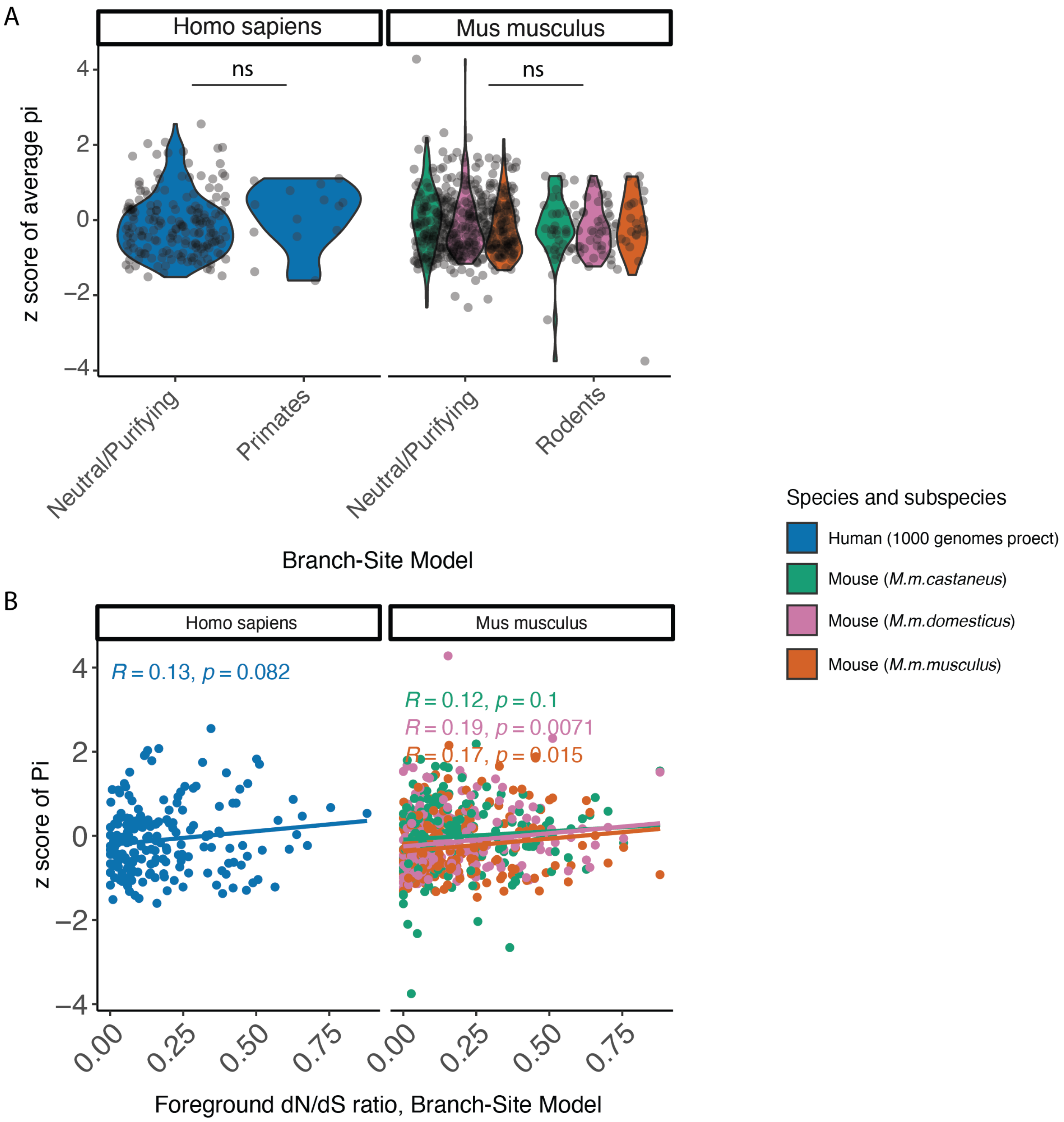

Supplementary Figure 2: (A) Distribution of the normalized pairwise sequence divergence, π, across genes evolving via neutrality or purifying selection versus genes harboring signals of clade-specific adaptive protein evolution. The left panel features data from the primate branch-site model, whereas the right panel displays results from the rodent branch-site model. (B) Pearson correlation between the dN/dS estimated under the Branch-Site Model for each group (primates and rodents) and the normalized pairwise sequence divergence (π) in human and house
